# Cardiac Allograft Rejection Induces Changes in Nucleocytoplasmic Transport: RANGAP1 as a Potential Non-Invasive Biomarker

**DOI:** 10.3390/jpm12060913

**Published:** 2022-05-31

**Authors:** Silvia Lozano-Edo, Esther Roselló-Lletí, Ignacio Sánchez-Lázaro, Estefanía Tarazón, Manuel Portolés, Maryem Ezzitouny, Raquel Lopez-Vilella, Miguel Angel Arnau, Luis Almenar, Luis Martínez-Dolz

**Affiliations:** 1 Heart Failure and Transplantation Unit, Cardiology Department, University and Polytechnic La Fe Hospital, 46026 Valencia, Spain; ignaciosanchezlazaro@gmail.com (I.S.-L.); mezzitouny@gmail.com (M.E.); cune10@hotmail.com (R.L.-V.); shattowsky@gmail.com (M.A.A.); lualmenar@gmail.com (L.A.); luismartinezdolz@gmail.com (L.M.-D.); 2 Clinical and Translational Research Group in Cardiology, Health Research Institute Hospital La Fe (IIS La Fe), 46026 Valencia, Spain; esther_rosello@iislafe.es (E.R.-L.); tarazon_est@gva.es (E.T.); portoles_man@gva.es (M.P.); 3 Center for Biomedical Research Network on Cardiovascular Diseases (Centro de Investigación Biomédica en Red de Enfermedades Cardiovasculares: CIBERCV), 28029 Madrid, Spain

**Keywords:** biomarkers, RANGAP1, cardiac rejection, heart transplantation, nucleocytoplasmic transport

## Abstract

The non-invasive diagnosis of acute cellular rejection (ACR) is a major challenge. We performed a molecular study analyzing the predictive capacity of serum RanGTPase AP1 (RANGAP1) for diagnosing ACR during the first year after heart transplantation (HT). We included the serum samples of 75 consecutive HT patients, extracted after clinical stability, to determine the RANGAP1 levels through ELISA. In addition, various clinical, analytical, and echocardiographic variables, as well as endomyocardial biopsy results, were collected. RANGAP1 levels were higher in patients who developed ACR (median 63.15 ng/mL; (inter-quartile range (IQR), 36.61–105.69) vs. 35.33 ng/mL (IQR, 19.18–64.59); *p* = 0.02). Receiver operating characteristic (ROC) curve analysis confirmed that RANGAP1 differentiated between patients with and without ACR (area under curve (AUC), 0.70; *p* = 0.02), and a RANGAP1 level exceeding the cut-off point (≥90 ng/mL) was identified as a risk factor for the development of ACR (OR, 6.8; *p* = 0.006). Two independent predictors of ACR identified in this study were higher RANGAP1 and N-terminal pro-brain natriuretic peptide levels. The analysis of the ROC curve of the model showed a significant AUC of 0.77, *p* = 0.001. Our findings suggest that RANGAP1 quantification facilitates risk prediction for the occurrence of ACR and could be considered as a novel non-invasive biomarker of ACR.

## 1. Introduction

Cardiac rejection is a major cause of allograft loss and mortality [1]. The detection of cardiac rejection using non-invasive methods continues to be a major challenge. To date, the histological evaluation of endomyocardial biopsy (EMB) remains the gold standard for the diagnosis of acute cellular rejection (ACR). However, it is an invasive procedure associated with several risks [2,3]. Currently, the determination of troponin, natriuretic peptide, calcium regulator, microRNA, cell-free DNA, and inflammatory marker levels are the most studied non-invasive methods for monitoring ACR; however, none of them provide accuracy comparable to EMB [4,5,6,7,8,9,10].

In this study, our team has investigated the influence of cellular rejection on nucleolar organization in patients undergoing heart transplantation (HT). The nucleus plays a fundamental role in the general functioning of the cell [11]. Previous studies have shown that patients with HF present alterations in the nucleocytoplasmic trafficking machinery, leading to increased levels of importins, exportins, Ran regulators, and nucleoporins, as well as alterations in calcium metabolism [12,13,14]. Previously, our group observed alterations in the nucleocytoplasmic trafficking machinery components in patients with cardiac rejection in a small group of patients (Lozano-Edo et al., 2021) [15]. Since cardiac rejection produces inflammation, disintegration, and cell necrosis [16], we hypothesized that it can also alter nucleocytoplasmic transport, which would result in changes in the circulating serum levels of the nuclear pore complex components. Specifically, changes are anticipated in the Ran regulatory system, which we could detect in patient serum in a relatively simple and non-invasive way.

Therefore, due to its crucial role in protein export from the nucleus to the cytoplasm and regulation of the nuclear pore complex, we analyzed the expression of RANGAP1 in HT patients. The objective of this study was to determine whether the increase in circulating levels of RANGAP1, once the patient reaches the period of clinical stability, i.e., 1–3 months after transplantation, could serve as a marker for predicting ACR during the first year of follow-up [17,18,19].

## 2. Methods

### 2.1. Collection of Samples

In this prospective study, 75 adult patients who underwent HT at our center were consecutively enrolled over a 3-year period from January 2017 to December 2019. Serum samples were collected from all patients once the period of clinical stability was reached, between 1–3 months after HT. The average timing of sampling was 1.9 ± 0.9 months. A single sample was avaible from each patient. Simultaneously, EMB results were collected prospectively to document the rejection episodes that occurred in each patient during the first year of follow-up. In routine clinical practice, EMB is performed to detect rejection at 1, 2, 3, 4, 6, 9, and 12 months after HT. Patients maintained a standard immunosuppression regimen, and rejection episodes were assessed according to the International Society for Heart and Lung Transplantation (ISHLT) consensus report [16]. EMB histology was performed by an expert pathologist blinded to clinical and laboratory information. Various clinical characteristics of transplant patients, including age, sex, body mass index (BMI), primary heart disease, biochemical markers (high-sensitivity troponin T and N-terminal pro-brain natriuretic peptide (NT-proBNP)), echocardiographic parameters, and immunosuppressive drug regimen, were recorded during the first year after transplantation.

Moreover, patients who died in the first months after HT, without reaching clinical and hemodynamic stability, were excluded, considering that mortality in this period fundamentally occurs due to surgical complications and primary graft dysfunction. Similarly, patients undergoing cardiopulmonary transplantation, heart retransplantation, those whose blood samples were not available for the analysis of RANGAP1, and those who did not sign the informed consent prior to extraction were excluded.

### 2.2. Measurement of Circulating RANGAP1

Blood samples were obtained using peripheral venipuncture via a 10 mL glass vacuum extraction tube, treated with 15% EDTA anticoagulant (0.12 mL) (BD Vacutainer^®^ K3E; REF 368480). The tubes were centrifuged (Eppendorf 5415R, Eppendorf, Germany) at 1300 rpm for 10 min at 4 °C, and the supernatant was collected and aliquoted into 500 μL screen-printed plastic cryotubes, which were subsequently stored in the biobank of La Fe University Hospital (Valencia, Spain) at −80 °C until further analysis. The assay was performed on all samples at the same time.

RANGAP1 expression was determined using a specific ELISA kit (cat. MBS9321016; MyBiosurce Inc., San Diego, CA, USA). The RANGAP1 test has a detection limit of 1.00 ng/mL and sensitivity of 1.00 ng/mL; both intra-assay CV (%) and inter-assay CV (%) are less than 15%.

### 2.3. Measurement of Circulating NT-proBNP and High-Sensitivity Troponin T

Peripheral blood samples were collected in the first months after heart transplantation (once the period of clinical stability had been reached) and 12 months after it, in order to determine the levels of NT-proBNP and high-sensitivity troponin T. NT-proBNP measurement was performed by chemiluminescent microparticle immunoassay (CMIA) (analyzer; Alinity i; trading house: Abbott^®^), and high-sensitivity troponin T measurement was performed by electrochemiluminescence immunoassay (ECLIA) (analyzer: Cobas e; commercial house: Roche^®^).

### 2.4. Statistical Analysis

Descriptive analyses were performed for all reference variables, and the results were expressed as mean and standard deviation (SD), median and interquartile range (IQR) for continuous variables, and as number and percentage for discrete variables. The results for each variable were tested for normality using the Kolmogorov–Smirnov method. Differences between groups were analyzed using Student’s t-test for independent samples and the chi-square test. Continuous variables that did not follow normal distribution were compared using the Mann–Whitney U test.

The sensitivity and specificity of plasma RANGAP1 levels for ACR detection were assessed by plotting receiver operating characteristic (ROC) curves. Logistic regression was performed to evaluate the contribution of circulating RANGAP1 in combination with age, sex, BMI, hemoglobin, creatinine, and logarithm (log) of troponin and Nt-proBNP determined in the period of clinical stability, which were included in the model for predicting ACR. Differences were considered statistically significant at *p* < 0.05. All statistical analyses were performed using SPSS version 25.0 (IBM Corp., Armonk, NY, USA).

## 3. Results

### 3.1. Study Population and ACR

A total of 75 HT recipients were included in this analysis. The mean (±SD) age of the patients was 52 ± 14 years, and men constituted 79% of the cohort. The patients were divided into two groups: those who did not present clinically relevant ACR (no rejection or mild rejection (grade 1R)) during the first year after HT (*n* = 59), and those who presented clinically relevant ACR (moderate or severe rejection (grades 2R or 3R)) (*n* = 16). Of the 16 patients who presented ACR 2R–3R, 68.75% presented it in the first 4 months after HT, 12.5% between 4 and 8 months after HT, and 18.75% between 9 and 12 months after transplantation. In patients who presented rejection ≥ 2R in the first four months after HT, the RANGAP1 sample was extracted at a mean of 1.82 ± 0.98 months after HT. In patients who presented rejection ≥ 2R between the fourth and the eighth month after HT, the RANGAP1 sample was extracted, on average, at 1.50 ± 0.71 months after HT. Finally, in patients who presented ACR ≥ 2R between the nineth and twelfth month after HT, RANGAP1 levels were obtained, on average, at 1.33 ± 0.58 months after HT. In the group of patients with ACR ≥ 2R, the mean time between the HT and the extraction of the sample for the RANGAP1 analysis was 1.69 ± 0.87 months.

As shown in Table 1, both groups of patients displayed similarities in terms of clinical and demographic variables, i.e., sex, age, BMI, presence of diabetes, hypertension, and dyslipidemia prior to HT, and subsequent immunosuppressive regimen. Furthermore, significant differences were not observed in ventricular function between patients with clinically relevant rejection and those who did not show ACR during the first three months after HT (stability period). However, 12 months after HT, a higher percentage of ventricular dysfunction was observed in the group of patients who presented ACR during the first year after transplantation (13.3% vs. 1.7%; *p* = 0.05) (Table 1).

Similarly, statistically non-significant trends were observed when the group of patients with clinically relevant ACR and those who did not present clinically relevant ACR were compared based on NT-proBNP (1499 pg/mL (798–2841) vs. 2070 pg/mL (757–7825), *p* = 0.23) and troponin levels (68.76 ng/L (42.7–186) vs. 87.93 ng/L (56.1–139), *p* = 0.31) in the first months after HT. Since NT-proBNP and troponins did not present normal distribution in our analysis, we performed logarithmic transformation of their values to reveal higher levels of both categories of biomarkers in the group of patients with clinically relevant ACR, bordering on statistical significance in the case of log NT-proBNP (3.42 (0.64) vs. 3.18 (0.45), *p* = 0.09)). The analytical parameters are presented in Table 2.

### 3.2. RanGAP1 Analysis in ACR

**Figure 1:**
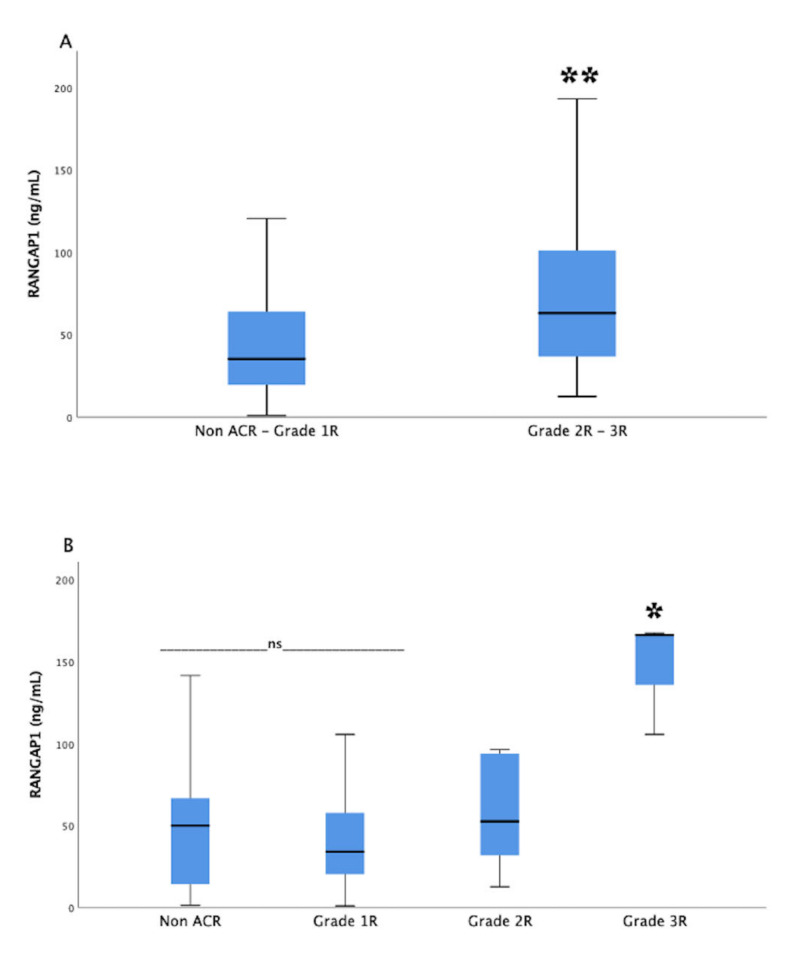
Circulating levels of RANGAP1 between normal and rejection heart allografts. Comparison between the serum levels of RANGAP1 in patients without and with significant acute cell rejection (ACR) (**A**). Comparison between the serum levels of RANGAP1 across different grades of rejection in heart allografts (**B**). The middle line in the box plots represents the median. The lower box represents the first quartile. The upper box represents the third quartile. Whiskers indicate the 95% confidence interval of the mean. ** *p* = 0.02, * *p* = 0.03. Grade 1R, mild rejection; grade 2R, moderate rejection; grade 3R, severe rejection; ns, no statistically significant differences.

The serum RANGAP1 levels were higher in patients with significant ACR (2R–3R) compared to the group of patients without significant ACR (median 63.15 ng/mL (IQR, 36.61 to 105.69] vs. 35.33 ng/mL (IQR, 19.18 to 64.59); *p* = 0.02) (Figure 1A). When we compared RANGAP1 levels in patients without ACR to those with mild rejection (1R), we found no significant differences (mean 50.11 ng/mL (IQR, 11.11 to 74.2) vs. 34.04 ng/mL (IQR, 20.00 to 60.71); *p* = 0.98). In contrast, significantly higher RANGAP1 levels were observed in the group of patients with severe rejection compared to those with moderate rejection (mean 166.33 ng/mL (IQR, 105.69 to 166.33) vs. 52.60 ng/mL (IQR, 29.48 to 94.38); *p* = 0.03) (Figure 1B).

**Figure 2:**
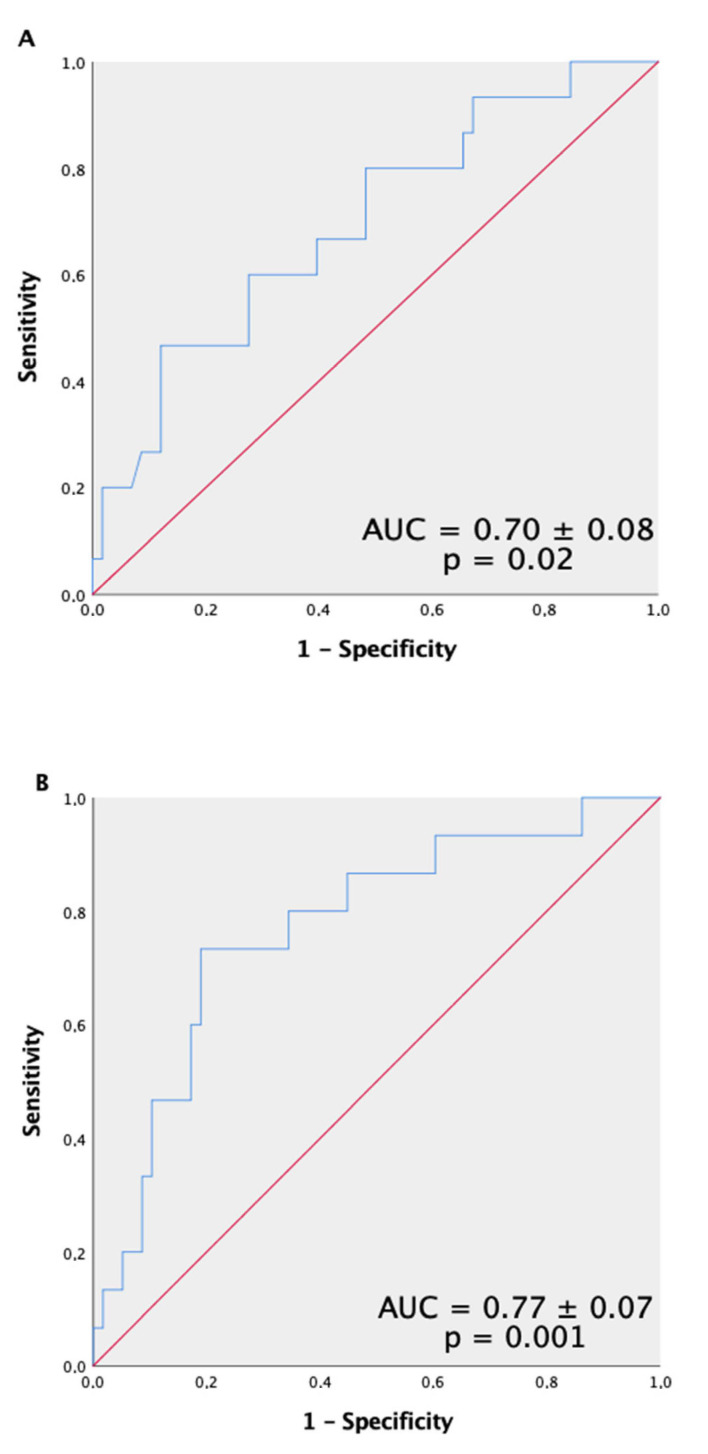
Receiver operating characteristic (ROC) curve of circulating RANGAP1 for the detection of cardiac allograft rejection. Ability of RANGAP1 to detect significant ACR (grades 2R–3R) (**A**). The two independent predictive blood parameters indicative of significant ACR: RANGAP1 and log NT-proBNP combined (**B**).

We constructed receiver operating characteristic (ROC) curves to determine the ability of RANGAP1 to detect ACR, obtaining a significant area under the curve (AUC, 0.70 ± 0.08 (95% confidence interval (CI) 0.55 to 0.85); *p* = 0.02) (Figure 2A), with an optimal cut-off point of 90 ng/mL (sensitivity, 47%; specificity, 86%; positive predictive value, 45.6%; and negative predictive value, 86.6%). We observed that in patients with RANGAP1 levels >90 ng/mL, 44% presented ACR in the first year after HT, while in those with RANGAP1 levels <90 ng/mL, only 14% presented ACR. Subsequently, we obtained an ROC curve of the log NT-proBNP, a variable that had bordered on statistical significance in our study; however, statistical significance was not obtained (AUC, 0.60 ± 0.09 (95% CI, 0.42 to 0.77); *p* = 0.24).

We then performed multivariate logistic regression analysis to determine whether circulating serum levels of RANGAP1 were independent predictors of ACR. Age, sex, BMI, serum RANGAP1 levels, creatinine, hemoglobin, and log of troponins and NT-proBNP determined during the clinical stability period were included in the model. The multivariate model revealed that a RANGAP1 value >90 ng/mL (optimal cut-off point determined by the ROC curve) was an independent predictor of ACR with an odds ratio (OR) of 6.8 (95% CI, 1.74–26.88; *p* = 0.006) and C-statistic of 0.79 ± 0.07 (95% CI 0.66–0.93) *p* = 0.001). The log of NT-proBNP values was also identified as an independent factor in the model (OR, 3.52; 95% CI, 0.97–12.71; *p* = 0.05). Furthermore, we constructed an ROC curve with the combination of the two independent predictive blood parameters of ACR, i.e., RANGAP1 and Log NT-proBNP, obtaining a significant AUC (AUC, 0.77 ± 0.07 (95% CI 0.64 to 0.91), *p* = 0.001) (Figure 2B).

## 4. Discussion

Our study proposes that since nucleocytoplasmic transport is altered in patients suffering from ACR after HT, the determination of RANGAP1 levels could be useful in predicting ACR during follow-up, as a non-invasive biomarker of rejection. We found significantly higher serum levels of RANGAP1 in patients with ACR, suggesting that a serum RANGAP1 level >90 ng/mL is an independent predictor of ACR.

The conventional “gold standard” of rejection, EMB, is associated with sampling errors and inter-observer variability [3], and since it is an invasive procedure, there are potential complications for the patient [2]. Owing to these limitations and risk factors, many studies have focused on investigating non-invasive techniques to provide additional information for predictions after HT. The study of cardiac biomarkers capable of predicting cardiac rejection has gained substantial interest. Previous studies have reported alterations in calcium metabolism in patients with cellular rejection and primary graft dysfunction, with lower levels of Serca2a in patients who present these complications after HT [5,20]. In addition, perinuclear changes have been observed in biopsies of patients with cellular rejection [21]. Furthermore, ACR episodes have been associated with elevated levels of circulating troponin [6], and especially B-type natriuretic peptide, in addition to NT-proBNP [6,22,23,24]. Our results are in accordance with these studies, and we observed that NT-proBNP level was an independent predictor of ACR during the first year of follow-up in the multivariate model. Even though therapeutic strategies have advanced in recent years, substantial mortality is recorded in this group of patients [25]. However, studies presenting fundamental research on this subject remain limited. Therefore, research aimed at improving the ability to identify dysregulations in myocardial biology and to formulate potential treatments to reverse, prevent, or predict ACR is important.

Our results showed that circulating levels of RANGAP1 were able to distinguish patients with clinically relevant ACR, during the first year of follow-up, from those without this complication (AUC = 0.70), with an optimal cut-off point of 90 ng/mL. We observed that the log of NT-proBNP level was higher in the group of patients with clinically relevant ACR (2R-3R), bordering on statistical significance. However, consistent with previous studies [26], our study shows that NT-proBNP in isolation does not have a high discriminative capacity to differentiate between patients with and without cardiac rejection. Our results verified RANGAP1 and NT-proBNP as independent predictors of ACR, which display a complementary value for the prediction of rejection and, thus, increase the detection capacity with an AUC of 0.77. Based on these results, we obtained a very good negative predictive value and an acceptable positive predictive value.

Even though alterations in nucleocytoplasmic transport have been discovered in patients with HF, with increased levels of RANGAP1 [12], these changes have not been analyzed for ACR to date. The role of immune response signaling in the regulation of the nuclear pore complex remains poorly understood. Previous studies have shown that the activation of T cells promotes the activation of RanGAP1, resulting in an increase in nuclear pore activity [27]. These findings are consistent with the results of our study, which reveal increased RanGAP1 levels in the group of patients with ACR involving the activation of T lymphocytes. Since the biological role of RanGAP1 in the pathophysiology of this process remains unexplored, our study presents a preliminary analysis that did not investigate the mechanistic insights into the relationship between RANGAP1 dysregulation and ACR. Nevertheless, a loss of myocytes has been reported in ACR, similar to left ventricular remodeling in heart failure, wherein myocyte necrosis or apoptosis leads to fibrosis [28]. Consequently, an increase in the activity of the nucleo-cytoplasmic machinery is required for de novo protein synthesis, thereby necessitating the overexpression of the Ran regulatory system [12]. Other immunohistochemical studies carried out in the smooth muscle of coronary and carotid arteries have observed that RANGAP1 levels are low in uninjured differentiated cells, while neointimal proliferation is associated with a significant increase in RANGAP1 levels [29]. Cardiac rejection leads to vascular involvement and damage, along with neointimal proliferation [30], in consistence with our findings.

The indicators identified in this study offer the possibility of detecting individuals with a high risk of ACR at an early stage during the first year of follow-up. However, more multicenter trials are needed to validate the use of this entity. The preliminary findings of our study need to be validated in larger cohorts to facilitate the use of this biomarker for substantial improvement in the surveillance strategy for cardiac rejection as a complement to EMB.

Our study had several limitations, and the results must be interpreted accordingly. This investigation involved only a single center, and included a relatively limited number of patients. Thus, the potential variability in serum levels of RANGAP1 must also be considered based on other parameters that were not analyzed in the present study, such as patient population and stress situations. In addition, our study focused on ACR and did not specifically evaluate antibody-mediated rejection (AMR). However, our findings provide important information for the prediction of ACR, which may be further supported by addressing the limiting factors in future studies. As this prospective study represents the observations from a single center, the results are relatively homogenous regarding the diagnostic and therapeutic strategies for these patients. This study is the continuation of the first study published by our research group (Lozano-Edo et al., 2021) in which we showed the initial results [15].

## 5. Conclusions

Patients with at least a moderate degree of ACR during the follow-up after HT showed higher serum levels of RANGAP1. This variable was found to be an independent predictor of ACR, and assessing its concentrations in combination with other variables, such as NT-proBNP, will possibly increase its predictive capacity for ACR. This combination is proposed as an effective tool for predicting ACR to facilitate decision making and individualized management of these patients. However, these preliminary findings need to be validated in larger prospective cohorts. Overall, RANGAP1 is a potential non-invasive biomarker of ACR.

## Figures and Tables

**Table 1:** Recipient characteristics, immunosuppressive therapy and echo-Doppler study.

Variables ^a^	Total (*n* = 75)	Non-Rejection—Grade 1R (*n* = 59)	Grades 2R–3R Rejection (*n* = 16)	*p*
**Clinics and demographics**				
Age, years	52.3 (14.0)	51.88 (14.5)	53.7 (12.3)	0.69
Male sex (%)	59 (78.7)	48 (81.4)	11 (68.8)	0.28
Body mass index (kg/m^2^)	25.48 (3.67)	25.48 (3.87)	25.46 (3.04)	0.99
Hypertension (%)	24 (32.9)	20 (35.1)	4 (25.0)	0.45
Diabetes mellitus (%)	8 (11.0)	6 (10.5)	2 (12.5)	0.82
Dyslipemia (%)	20 (27.4)	14 (24.1)	6 (40.0)	0.22
Ejection fraction pre-HT (%)	25.13 (15.64)	24.36 (16.42)	28.0 (12.37)	0.41
**Indication for cardiac transplantation** **0.73**				
Ischemic cardiomyopathy (%)	18 (24.0)	13 (22.0)	5 (31.3)	
Idiopathic dilated cardiomyopathy (%)	22 (29.3)	18 (30.5)	4 (25.0)	
Other (%)	35 (46.7)	28 (47.5)	7 (43.8)	
**Immunosuppressive therapy**				
Tacrolimus (%)	72 (96)	56 (94.9)	16 (100)	0.36
Mycophenolic acid (%)	71 (94.7)	55 (93.2)	16 (100)	0.28
Steroids (%)	75 (100)	59 (100)	16 (100)	
**Echo-Doppler study post-HT**				
Moderate/severe ventricular dysfunction (%) 1–3 months	2 (2.9)	1 (1.7)	1 (6.3)	0.32
Moderate/severe ventricular dysfunction (%) 12 months	3 (4)	1 (1.7)	2 (13.3)	0.05
Moderate/severe pericardial effusion (%) 1–3 months	8 (10.7)	5 (8.5)	3 (18.8)	0.24
Moderate/severe pericardial effusion (%) 12 months	2 (2.9)	2 (3.6)	0 (0)	0.45
Moderate/severe left ventricular hypertrophy (%) 1–3 months	7 (9.3)	5 (8.5)	2 (12.5)	0.62
Moderate/severe left ventricular hypertrophy (%) 12 months	0 (0)	0 (0)	0 (0)	

^a^ Categorical data are presented as number (%) and continuous data as mean (SD). The *p* value was obtained by comparing non-rejection grade 1R with rejection grades 2R–3R. Grade 1R, mild rejection; grade 2R, moderate rejection; grade 3R, severe rejection; HT, heart transplantation.

**Table 2:** Laboratory.

Variables ^a^	Total (*n* = 75)	Non-Rejection—Grade 1R (*n* = 59)	Grades 2R–3R Rejection (*n* = 16)	*p*
NT-proBNP (pg/mL) 1–3 months	1523 (798–3548)	1499 (798–2841)	2070 (757–7825)	0.24
Log NT-proBNP (pg/mL) 1–3 months	3.23 (0.50)	3.18 (0.45)	3.42 (0.64)	0.09
NT-proBNP (pg/mL) 12 months	341 (215–586)	334 (216–540)	460 (211–1066)	0.31
Log NT-proBNP (pg/mL) 12 months	2.60 (0.48)	2.53 (0.36)	2.82 (0.73)	0.15
Troponin T (ng/L) 1–3 months	7134 (47.78–159)	68.76 (42.73–186)	87.93 (56.18–139)	0.31
Log Troponin T (ng/L) 1–3 months	1.95 (0.47)	1.92 (0.46)	2.06 (0.51)	0.29
Troponin T (ng/L) 12 months	17.58 (8.39–33.36)	17.03 (8.68–33.93)	19.90 (8.04–37.34)	0.90
Log Troponin T (ng/L) 12 months	1.25 (0.34)	1.24 (0.33)	1.27 (0.37)	0.78
Hemglobine 1–3 months	11.25 (1.83)	11.45 (1.85)	10.54 (1.62)	0.08
Hemglobine 12 months	12.8 (1.71)	12.95 (1.59)	12.27 (2.11)	0.17
Creatinine 1–3 months	1.07 (0.51)	1.06 (0.56)	1.09 (0.31)	0.89
Creatinine 12 months	1.13 (0.33)	1.12 (0.33)	1.18 (0.32)	0.54

^a^ Continuous data are presented as mean (SD) and continuous variables with an abnormal distribution as median (interquartile range). Variables with non-normal distribution were converted to logarithms to obtain normal distribution. The *p* value is obtained by comparing non-rejection grade 1R with rejection grades 2R–3R. Grade 1R, mild rejection; grade 2R, moderate rejection; grade 3R, severe rejection.

## Data Availability

The data presented in this study are available on request from the corresponding author. The data are not publicly available due to ethical reasons.
